# Friendship and End-of-Life Discussions Among Older Adults: Moderating Effects of Marital Status and Gender

**DOI:** 10.1093/geroni/igaf122.149

**Published:** 2025-12-31

**Authors:** Zheng (Noah) Lian, Lucie Kalousova

**Affiliations:** Vanderbilt University, Nashville, Tennessee, United States; Vanderbilt University, Nashville, Tennessee, United States

## Abstract

Sharing healthcare preferences with others reduces the likelihood of unwanted treatments at the end-of-life (EOL), a key indicator of a “good death.” Spouses and other romantic partners are key facilitators of EOL planning decision-making, but little is known about the role of broader social networks, which may be particularly important for people who are single in the final years of their lives. This study investigates the association between emotional support from friends, friendship network size, and informal and formal EOL planning using data from the 2018 and 2020 waves of the Health and Retirement Study (HRS)—a nationally representative sample of U.S. older adults (N = 4,728). We use multivariable logistic regression models and evaluate how the associations vary by gender and marital status. Our findings reveal that emotional support from friends is differentially related to older adults’ engagement in EOL conversations. Among married, partnered, or previously married individuals, support from friends has no statistically significant effect on the likelihood of EOL discussion. We find evidence of moderation by marital status and gender. Older men with low emotional support are 68 percent less likely to engage in EOL discussions compared to those with high levels of support. Never married older men with strong emotional support from friends are as likely to have had informal EOL conversations as their married/partnered counterparts. These findings contribute to the literature by highlighting the critical role of high-quality friendships in EOL preparations for never married men.

